# Performance of students with different accessibility needs and preferences in “Design for All” MOOCs

**DOI:** 10.1371/journal.pone.0299090

**Published:** 2024-03-07

**Authors:** Alejandro Rodriguez-Ascaso, Elisa M. Molanes-López, Jorge Pérez-Martín, Emilio Letón

**Affiliations:** 1 Department of Artificial Intelligence, Universidad Nacional de Educación a Distancia (UNED), Madrid, Spain; 2 Department of Statistics and Operations Research, Faculty of Medicine, Universidad Complutense de Madrid (UCM), Madrid, Spain; COMSATS University Islamabad, PAKISTAN

## Abstract

Recent research has shown that Massive Open Online Courses (MOOCs) create barriers for students with disabilities. Not taking into account their needs in the design, production or delivery of MOOCs may be one of the main causes behind this. It leads to poor compliance with suitable learning designs and web accessibility standards, as well as a lack of knowledge about the students’ needs. The objective of our research is to analyze the learning performance of the students in MOOCs on topics related to Design for All, offered in an Open edX-based platform. Accessibility support was conceived from the outset, including compliance of both the platform and the learning resources with the WCAG 2.1 accessibility standard, and with a subset of the principles of Universal Design for Learning. Additionally, students were consulted on their accessibility needs and preferences, following publicly available modeling schemes and previous research. From a sample of 765 students, who completed at least one of the graded assessment activities of the course, a multilevel multiple logistic regression model was fitted. Based on that model, the results indicate that: a) users of screen readers and users of captions show a statistically significant positive association with a good performance when compared to students with no preferences, with an odds ratio of, respectively, OR = 13.482 and OR = 13.701; b) students who have low vision or very low vision show a significant negative association with a good performance when compared to users of screen readers and to users of captions, with OR = 26.817 and OR = 27.254, respectively.

## 1 Introduction

Massive Open Online Courses (MOOCs) are online, open and free-of-charge courses offered through the web to a massive audience. Like other online courses, MOOCs can take advantage of the web capabilities to deliver different kinds of static and dynamic content such as text, images, and videos, as well as interactive learning activities that can be accessed anytime, from anywhere, ideally. However, research has shown that MOOCs are not designed, produced or delivered considering the needs of students with disabilities [1–3]. The present paper aims to analyze the performance of a group of students with different accessibility needs and preferences in a set of MOOCs. Within the next subsections, different aspects of the accessibility of MOOCs are addressed. The final subsection defines the specific research questions and describes the structure of the present paper.

### 1.1 The framework of MOOC accessibility: UDL and WCAG

The IMS Global Learning Consortium defined accessibility as ‘the ability of the learning environment to adjust to the needs of all learners’, including those with disabilities, and this is determined by ‘the flexibility of the education environment and the availability of adequate alternative-but equivalent content and activities’ [4]. According to [5] the Universal Design for Learning (UDL) framework applies design principles to the science of how people learn, promoting the creation of materials that work for all learners, rather than a single, one-size-fits-all solution. UDL considers three principles: Provide multiple means of engagement, Provide multiple means of representation, Provide multiple means for action and expression [6]. On the other hand, the web is the universal, technological substrate where elearning takes place. Web accessibility standards, i.e., the Web Content Accessibility Guidelines (WCAG, [7]) constitute the most useful and universally accepted tool to check whether a specific web content is accessible to people with disabilities. These guidelines can be applied in any field where digital interactions occur: banking, mass-media, transports, education, etc. This is the reason why WCAG are referenced by legislative systems across the world (e.g. Section 508 in the United States, Web Accessibility Directive in Europe), as the reference standard for web accessibility, either directly or through other pieces of standardization. Furthermore, WCAG principles (perceivable, operable, understandable, and robust) are deeply connected to UDL guidelines [8]. However, accessibility is more than just a checklist for compliance. Understanding learners, how they interact with digital resources, and what learning strategies are applicable in each case (UDL, in the end), are field-specific aspects that should be necessarily considered when designing and developing truly accessible elearning, in addition to mere WCAG techniques. In other words, WCAG compliance is necessary but needs to be grounded in the UDL framework in order to address the varying needs of all learners [9]. Several studies consider UDL as the general framework for accessibility in open learning [10, 11]. The approach by [1] considers each of the phases of MOOC learning where accessibility should be considered: 1) pre-emptive information about the accessibility of course learning activities and resources should be included; 2) the accessibility of the platform as well as of the learning contents and activities should be ensured; 3) the use of accessible software and any other external resources involved in the activities should be allowed; 4) deadlines for submitting assignments should be flexible. Furthermore, accessibility support services should be provided during the run-time stage of courses.

### 1.2 Accessibility of MOOCs for different user groups

Research has shown that MOOCs often present technical accessibility barriers for students with disabilities when platforms and contents do not properly comply with WCAG. In 2013 and 2014 [12, 13], evaluated the accessibility of the Coursera platform and several of its courses, focusing, in the first year, on the needs of elderly students and the following year, on those who where visually impaired. In both studies, accessibility issues were reported. [14] evaluated the accessibility of basic tasks (platform registration, selection and enrollment in a course) by a blind person in several MOOC platforms, namely Udacity, Coursera, edX, OpenCourseWorld and Iversity. Although none of the platforms were fully accessible for a blind person, edX was the only one that allowed the fulfillment of all the tasks. In [15], eight MOOC platforms were reviewed, accessibility problems were reported, and edX and FutureLearn came out as the ones with less accessibility barriers. Attending MOOCs through mobile devices presents accessibility barriers, according to [16], who conducted tests with visually impaired users (on edX, Coursera, and Khan Academy) and presented a heuristic walk-through performed by five experts (on Coursera). However, for many user groups, MOOC accessibility has not been addressed yet, and therefore there is a need to focus research on case studies with particular accessibility needs in order to understand those needs in-depth [17]. Furthermore, beyond the technical assessment of compliance with WCAG, a more holistic, UDL-based framework for evaluating MOOC accessibility is needed. Current research [18] reports efforts for an iterative process to build such a framework in collaboration with students.

### 1.3 Institutional support: Disclosure of disability and personalized accessibility needs

Beyond WCAG-related accessibility barriers, literature reveals that MOOCs are not designed, produced or delivered considering the needs of users with disabilities [1, 2]. Well established frameworks in online learning [19, 20] recommend the assessment of the students’ accessibility needs, the provision of psycho-pedagogical support and adaptations, as well as the provision of support for choosing, buying or hiring appropriate support products. Institutional management of that support necessarily entails finding out what the needs and preferences of your students are, being aware of how well your resources suit the students’ needs, and providing them with suitable resources when they are not available. In line with this [21, 22], evidenced that disclosure and use of accommodations are tied to academic success in postsecondary education (PSE). [23] performed a systematic review of 36 studies conducted in the US, UK, Canada, Belgium, Norway and Ireland in order to understand the barriers and facilitators of disability disclosure in post-secondary education. Barriers to disability disclosure and requests for accommodations in PSE included stigma, discrimination, lack of knowledge of supports and how to access them, type of course and instructor, coping styles, and nature of the disability. Facilitators included supports and resources, coping and self-advocacy skills, mentorship, and realising the benefits of disclosure. In the MOOC realm, and according to [3], the identification of accessibility needs is a requirement for improving MOOC platforms and contents. During the admission process, formal online education institutions offer that their students declare any disabilities that might require accommodations for their studies, e.g. sight impairment, hearing impairment or dyslexia. Moreover, mature institutions provide support to students during this process in order to ensure the validity of this information, the appropriateness of accommodations, and also for economic reasons, since accommodations and fee exemptions may imply significant resource consumption for the organization [24, 25]. This is the case of the Open University of the UK, one of the biggest online universities in Europe, where students with disability are classified according to a checklist with 12 symptoms and medical conditions [25], namely: Blind or partially sighted; Deaf or hard of hearing; Restricted mobility; Restricted manual skills (difficulty handling items); Impaired speech; Dyslexia or other specific learning difficulties; Mental health difficulties; Personal care support; Fatigue or pain; Unseen disabilities (e.g. diabetes, epilepsy or asthma); Autistic spectrum disorder; Other disabilities. However, in MOOCs, the enrollment processes are less formal, students fill in very simple and straightforward registration and enrollment forms for platforms or courses. [26] demands the use of disability categories (e.g. visual impairment, hard of hearing or learning difficulties) in order to provide greater insight into differences within the population of learners with disabilities. Furthermore, understanding their needs when studying online requires more than knowing how many of them are enrolled in a particular course, or even what type of disability each of them has, as claimed by [27, 28]. The reason is that disability categories do not necessarily map with the needs and preferences each student has to study online, or with the appropriate techniques that elearning systems should implement to fulfill such needs and preferences. For example, persons with visual impairments may have completely different strategies to use a figure in the learning content of an online course, namely: 1) a screen reader to read out the alternative text, 2) a screen magnifier to enlarge considerably the figure’s size, 3) modifying browser or operating system options to adjust the size and contrast of objects displayed on the screen, 4) a combination of the strategies above. Even more, during her/his life, a single person may go through all these different accommodations as her/his personal needs change. The CreativeWork type by Schema.org [29] is based on previous work by IMS Global [30], which was also adopted as a family of ISO/IEC standards [31], and its evolution [4]. They define computer-readable vocabularies for describing both the accessibility needs and preferences of users, as well as the accessibility characteristics of digital learning resources. These vocabularies allow for the discoverability of resources that are suitable for a particular student’s needs and preferences. They ensure that user/machine agents can parse information to attend requests by humans or by other machines, and hence it may constitute an essential component of the system architecture supporting a holistic, data-driven management of digital accessibility in Learning Management Systems. Suitability of this standard for learning design tools has been supported in several research works like [32, 33]. Furthermore, several pieces of research have made use of this approach for designing content personalisation systems oriented to improve the accessibility of elearning [34, 35]. In [28], authors validated with more than 100 users a web form based on this schema. The form allowed students to describe their accessibility needs and preferences about the sensory options for accessing digital learning resources, and was part of a software module with user modeling and content personalisation services in an elearning architecture. In that work, authors suggested its potential application in MOOCs.

### 1.4 Attainment of students with disabilities in MOOCs

Concerning the uptake and attainment of students with disabilities in MOOCs [26, 36], argue that there are no published studies relating to the number of disabled learners taking up MOOCs, or their demographics, motivations, completion rates and satisfaction from MOOCs. In [37], authors conclude that in MOOC learners’ diverse intentions and motivations for enrollment influence their experiences within the course. In [26] authors analyzed data coming from learners enrolled in different MOOCs covering a range of subjects, from medicine and dentistry to physical sciences. According to their results, learners with disabilities are particularly interested in taking up MOOCs in order to determine if they can study at a higher educational level and to get flexible and free education. As far as we know, there are no published studies addressing the attainment of students with disabilities in MOOCs. In the case of online formal education, further research is needed to gain understanding about students with disabilities in post-secondary institutions, and how the use of online and distance education is impacting their success and degree attainment [38]. However, there are some pieces of work available that are summarized next. [25] compares the completion rate, the pass rate, and the grades obtained by students with and without disabilities who were taking courses at the Open University of the UK in 2009. He concluded that several groups of students with disabilities (namely, students with mental health difficulties, students with restricted mobility, students with unseen or other disabilities, and students with dyslexia or other specific learning difficulties) showed lower pass rates and poorer grades than non-disabled students. He also found that attainment was lower in some groups of students with multiple disabilities. In [27] authors analyzed 668 module presentations, again at the Open University, and they found out that in 80.4% of the module presentations there was a higher rate of non-disabled students who completed the module, while in 19.3% there was a higher rate of disabled students who completed the module. Similarly in Spain, a recent study [39] indicated that during the academic year 2018/2019 at UNED (Spanish distance learning university) 24.2% of the students with disabilities completed the master and degree online modules in which they were registered, whereas the percentage was 43.4% for all students.

### 1.5 Research questions and structure of the present study

Since the literature reports accessibility barriers that may hinder the learning of students with disabilities in MOOCs, and also a gap in the knowledge about their participation, engagement and attainment in open learning, we have analyzed the demographics and the learning performance of students in a set of MOOCs. The accessibility of the MOOCs had been ensured across the design, production, and delivery of the learning resources, in compliance with applicable recommendations and standards. In these MOOCs, students have described voluntarily their accessibility preferences and needs about the sensory options for accessing digital learning resources, through a survey based on a standardized schema.

The research questions of our work are:

- RQ1: what is the learning performance in MOOCs of students who declare to have any accessibility preferences or needs?
- RQ2: what are the differences in the learning performance of students with different accessibility preferences or needs?

To achieve our objective, the present paper has been structured as follows: first, in the current section we have reviewed the related literature and established the research questions. Second, we describe the methodology and the materials used in our research. Next, we present the results of our study and discuss them together with its possible limitations. Finally, we come to the conclusions derived and future work.

## 2 Materials and methodology

### 2.1 Materials

The four courses analyzed in our study were developed under the ‘ONCE Foundation Channel at UNED’ project. According to its mission, the project seeks to offer MOOC based accessible training on digital competencies and “Design for All” [40], among other goals. Furthermore, the project undertakes research activities to better understand current MOOC barriers for students with disabilities, and how to overcome them. The four courses were announced in the list of courses of UNED Abierta’s Open edX instance (https://iedra.uned.es/courses), which is the open education provider at UNED. These courses are open to anyone willing to enroll in them. A subset of the enrolled students in the four MOOCs took part in the voluntary surveys. These free and open courses are not part of the UNED’s formal education offer.

Within the courses, students interacted with graded learning activities and with voluntary surveys. Students were informed that their answers to voluntary surveys would support the project to develop further its educational and social mission, and that their anonymity will be preserved at all times. More specifically, every voluntary survey was introduced with the following statement: “The questions below are voluntary and do not count towards the course grade. By answering them, you will help us to further develop our educational and social work. The survey is anonymous and while the results may be made public, the anonymity of the respondent will be preserved at all times. Thank you very much!”. All participants in this study were adults (see Section 3.1, where the age distribution is detailed). The research ethics committee of our University certified that our work meets its minimum requirements. UNED is the largest campus in Europe, and it complies with European data protection regulation (GDPR) and applicable ethics standards. In particular, we, as authors of this paper, have followed the “Ethics and data protection” guidelines by the European Commission. The MOOCs which data are analyzed in this article are provided by UNED Abierta, the UNED department for open learning. Upon their registry in UNED Abierta, all students of the MOOCs analyzed in this paper have explicitly accepted the conditions of UNED Abierta’s Terms of Service, which purposes include the “study and experimentation of new methodologies and disruptive pedagogical strategies that make possible the use of technology and the internet”. Furthermore, UNED’s mission includes “Commitment to equality, excellence and scientific rigor steer our public service vocation”, which connects with our paper’s objective of describing performance of MOOC students with and without disabilities.

We analyzed one presentation of each of the four courses described below.

- ACC (ICT Accessibility in public procurement). This course introduces the European standard on ICT (Information and Communication Technologies) accessibility and its application in public procurement as an enabler of inclusiveness.
- MAT (Accessible digital materials). This course provides easy-to-use strategies and resources to create accessible digital materials by following best practices in the creation of images, texts and videos. that reach a broader audience, both with and without disabilities, causing a greater impact on the Internet, and complying with ethics and current legislation.
- MOV (Accessible mobile devices for all). This course focuses on describing the needs and preferences that people with different types of disabilities have for using a mobile device, as well as the accessibility functions that are usually available on Apple (iOS) and Android devices. Special emphasis is made for people with low vision and people with limited mobility.
- INT (Human-computer interaction). This course allows to understand and apply the principles of Human-Computer Interaction, namely operation, perception and understanding, by applying the perspective of Design for All and support products in ICT.

The courses’ instructional design combines videos, texts and images, graded self-assessment tests, voluntary peer-to-peer evaluation activities, as well as forums with teaching and tutorial attention. Course assessment is undertaken through quizzes (graded assessment activities), which results are averaged to yield the course grade for each student on a scale from 0 to 1.

In agreement with the projects’ commitment with course accessibility, the platform, contents, and related services, comply with level AA of the WCAG 2.1 Guidelines of the W3C, and with the European standard EN 301 549 on ICT accessibility requirements. Open edX provides an accessible platform that conforms to WCAG 2.1 AA, according to its accessibility statement. Regarding the course materials, they are produced from the beginning in compliance with WCAG 2.1 guidelines. Once materials are produced, a team member (who is an expert in WCAG and who has not been involved in the previous production process) is responsible for adding those materials to the MOOC space at Studio (the Open edX’s content authoring Web service) while checking their accessibility. Finally, all courses have been tested by users with different needs and preferences, and each course has an accessibility page where users are encouraged to report any accessibility or usability issues they might find. Only minor problems were reported, which were promptly addressed when they related to the accessibility of teaching materials.

Beyond compliance with WCAG 2.1 AA and EN 301 549, the set of course features that are aligned with UDL guidelines are listed in Table 1.

**Table 1:** Course features aligned with UDL guidelines (i.e., provide multiple means of either engagement, representation, or action and expression).

Feature	UDL Guideline
Course contents can be accessed either sequentially or by browsing the table of contents.	Physical Action
There is sufficient contrast between foreground and background colors in video materials.	Perception
Videos have captions, audiodescription and interpretation in Spanish sign language.	Perception
Videos have interactive transcripts, which allow users to click on a transcript fragment to go to the corresponding video part.	Physical Action
Captions and interactive transcripts of videos can be shown/hidden by users.	Perception
In videos, visual contents and their corresponding oral descriptions are temporally synchronized, that is, presented simultaneously rather than successively.	Recruiting Interest
Mathematical texts are encoded through MathML.	Language & Symbols
Glossaries are provided, with definitions of new and complex terms.	Comprehension
HTML embedded support is provided for acronyms and for non-Spanish content.	Comprehension; Language & Symbols
Illustrations are used as means to provide multiple representations of ideas and concepts.	Perception
Every module includes a prompt to share reflections on the course concepts with staff and peers in the forum.	Sustaining Effort & Persistence
Depending on the specific course, lecturers’ and tutors’ support through forum is provided for 2–5 weeks.	Sustaining Effort & Persistence
Depending on the course, courses are open from 4 to 6 months, beyond the period where forum support is provided.	Self Regulation
Activities are open for students during the whole duration of the course.	Self Regulation
Progress (overall and per activity) is represented for students both numerically and graphically.	Sustaining Effort & Persistence; Executive Functions
Self-pace strategies are acknowledged and supported.	Self Regulation
Pre-emptive information is offered about the average student’s dedication (3 hours a week).	Self Regulation
In addition to free enrollment, two certification schemes are offered.	Recruiting Interest
A course schedule is provided, with timing recommendations for each learning activity.	Self Regulation
Weekly emails inform students on the week’s learning activities, according to the schedule, including links to salient activities.	Sustaining Effort & Persistence; Self Regulation
Pre-emptive information about course accessibility is offered to prospective students.	Recruiting Interest
Specialized accessibility support is provided through email, forum, and specific course sections.	Self Regulation; Expression & Communication

All courses include voluntary surveys where students describe their demographics (gender, age group, education level, occupation, dedication, sector and number of previous MOOCs), and their accessibility needs and preferences. The survey where students reported their accessibility preferences with digital learning materials was based on the work described in [28], which was based on the vocabularies of [30, 31]. That work confirmed the survey’s utility to gather the information needed to attend each student’s needs. Furthermore, in that work the survey’s usability was optimized for students with different abilities, i.e., people with visual, hearing, or mobility impairments, as well as non-disabled users. The Accessibility Preferences form offers different sensory options for accessing digital learning resources, but it does not address other preferences related to their operation, such as mobility-related preferences. The form provides the following options:

A Adaptations of text (books, articles, notes, etc.)
  1. Spoken texts (texts that can be heard, such as Daisy books or spoken books)
  2. Increased font size
  3. Increased contrast between foreground and background
B Adaptations of audio (for example, radio programs)
  1. Sign language interpretation
C Adaptations of images (diagrams, graphs, pictures, etc.)
  1. Alternative text (text description that is equivalent to the image)
D Adaptations of animations (sequence of images that are reproduced sequentially to represent a moving scene, for example, an animation of the solar system):
  1. Alternative text (text description that is equivalent to the animation)
E Adaptations of films and videos (for example, a videoconference or a television program)
  1. Audiodescription (additional narration track, which consists of a narrator describing what is happening on the screen)
  2. Captions (text that appears in the lower part of the image transcribing the dialogues, describing the sounds, etc.)
  3. Sign language interpretation

Based on the students’ responses to the accessibility preferences form described above, we define the mutually exclusive groups listed below:

- Raw Group 1: Students who did not take the survey.
- Raw Group 2: Students who took the survey and declared to have no preferences.
- Raw Group 3: Blind students.
- Raw Group 4: Students with low vision.
- Raw Group 5: Students with very low vision.
- Raw Group 6: Students who are users of captions.
- Raw Group 7: Students who are users of sign language.
- Raw Group 8: Students who are users of captions and sign language.
- Raw Group 9: Students who have low vision and are users of captions.
- Raw Group 10: Students who have low vision and are users of sign language.
- Raw Group 11: Students with other preferences, i.e., students whose preferences do not fit in any of the above groups.

In Table 2, we detail the mapping of Raw Groups 2–10 to the the accessibility preferences form, according to the chosen and not chosen items (check and cross marks, respectively) form. A blank cell indicates that the row form item is irrelevant for the corresponding column Raw Group.

**Table 2:** Mapping of the Raw Groups 2–10, according to the items selected by students in the accessibility preferences form: ✓ (selected), × (non-selected), and blanks (non-relevant).

	Raw Group								
Form item	2	3	4	5	6	7	8	9	10
Spoken texts (A1)	×				×	×	×	×	×
Increased font size (A2)	×	×	✓	✓	×	×	×	✓	✓
Increased contrast (A3)	×	×		✓	×	×	×		
Alternative text (C1 or D1)	×	✓	×	✓	×	×	×	×	×
Audiodescription (E1)	×	✓	×	✓	×	×	×	×	×
Captions (E2)	×	×	×	×	✓	×	✓	✓	×
Sign language (B1 or E3)	×	×	×	×	×	✓	✓	×	✓

### 2.2 Statistical methods

According to the objective stated in the introduction, the outcome variable of interest is Grade (the Course grade, on a scale from 0 to 1), and the explanatory variables of interest are Course and Group. The normality assumption of Grade will be analyzed in each of the categories of the explanatory variables using Shapiro-Wilk test. In the case of normality, a multiple linear regression model will be explored. Otherwise, a multiple logistic regression model will be fitted based on a dichotomization of the Grade outcome variable that identifies the students with good performance using an appropriate threshold value for the Grade variable.

In any case, a multilevel multiple regression approach will be used to take into account the possibility that the same individual may take more than one course. Additionally, demographic variables that are significant in the bivariate analysis will be considered as explanatory variables to be included in the model.

## 3 Results

We will show in Subsection Basic descriptive analysis a basic descriptive analysis and in Subsection Multilevel multiple regression analysis a multilevel multiple regression approach. Given that in the regression analysis we will consider INT as a reference category for Course and Raw Group 2 for Group, these categories will be shown in first place in the results regarding the descriptive analysis.

### 3.1 Basic descriptive analysis

A total of 2714 students were enrolled in the four courses analyzed (400 in INT, 628 in ACC, 1446 in MAT, and 240 in MOV). Since we focus on student performance (measured in terms of Grade variable), it is important to note that only 765 students filled in at least one of the graded assessment activities of the course. In Table 3, we collect the mean, standard deviation (*SD*), median, and first and third quartiles (*Q*_1_ and *Q*_3_) of Grade per course for these 765 students. Additionally, we include in Table 3 the participation, i.e. the percentage of enrolled students that participate in the assessment of each course. It is important to note that the course with the highest percentage of participation was INT with 43%.

**Table 3:** Summary measures of Grade by course.

Course	Mean (*SD*)	Median (*Q*_1_,*Q*_3_)	n	Participation
INT	0.408 (0.328)	0.330 (0.090, 0.750)	170	43% (170/400)
ACC	0.648 (0.346)	0.825 (0.240, 0.940)	176	28% (176/628)
MAT	0.556 (0.350)	0.610 (0.180, 0.910)	327	23% (327/1446)
MOV	0.672 (0.324)	0.850 (0.313, 0.923)	92	38% (92/240)
Total			765	28% (765/2714)

Preliminary results show small sample sizes in several raw groups. Taking into account this fact, raw groups with similar accessibility characteristics were combined. This process resulted in eight final groups (groups, hereinafter), with Group 1 (Raw Group 2) as the reference category. The groups are listed next:

- Group 0 (Raw Group 1): Students who did not take the survey.
- Group 1 (Raw Group 2): Students who filled-in the survey and declared to have no preferences.
- Group 2 (Raw Group 11): Students whose accessibility preferences did not fit in any of the established groups.
- Group 3 (Raw Group 3): Blind students.
- Group 5 (Raw Group 4 + Raw Group 5): Students with low vision + students with very low vision.
- Group 6 (Raw Group 6): Students who are users of captions.
- Group 8 (Raw Group 7 + Raw Group 8): Students who are users of sign language + students who are users of captions and sign language.
- Group 10 (Raw Group 9 + Raw Group 10): Students who have low vision and are users of captions + students who have low vision and are users of sign language.

Table 4 shows the basic descriptive statistics of Grade per Group, together with each Group’s percentage of participation. As for the Grade, 0.75 will be used as the threshold value to define good performance, since it is 75% of the maximum grade (1.0), and it is close to the median grade of the reference group (Group 1). Therefore, grades over 0.75 indicate good performance.

**Table 4:** Summary measures of Grade by accessibility preferences group.

Group	Mean (*SD*)	Median (*Q*_1_,*Q*_3_)	n	Participation
1	0.617 (0.333)	0.770 (0.240, 0.890)	53	65% (53/81)
0	0.516 (0.358)	0.520 (0.140, 0.890)	388	18% (388/2177)
2	0.577 (0.344)	0.700 (0.220, 0.895)	255	70% (255/365)
3	0.820 (0.199)	0.860 (0.813, 0.915)	12	86% (12/14)
5	0.496 (0.390)	0.460 (0.133, 0.900)	22	85% (22/26)
6	0.872 (0.149)	0.910 (0.840, 0.970)	13	87% (13/15)
8	0.673 (0.330)	0.760 (0.535, 0.940)	15	54% (15/28)
10	0.696 (0.341)	0.900 (0.525, 0.930)	7	88% (7/8)
Total			765	28% (765/2714)

Regarding the demographic variables for these 765 students, we have the following:

- 296 are women, 214 are men, 255 are missing values.
- the age of 40 participants are between 18 and 25 years old, 121 between 26 and 35, 183 between 36 and 45, 136 between 46 and 55, 33 between 56 and 65, 3 are over 65, and 249 are missing values.
- 3 participants have primary level of education, 68 secondary level, 278 bachelor degree, 125 master degree, 40 PhD degree, and 251 are missing values.
- 51 are students, 289 are employed, 145 study and work, 29 neither study nor work, and 251 are missing values.
- 31 are full time students, 345 work full time, 70 work part time, 32 are looking for work, 12 are retired, 26 are in other situations, and 249 are missing values.
- 259 work in private sector, 210 in public sector, 9 in other sectors, and 287 are missing values.
- for 185 it was the first time they enrolled in a MOOC, 89 had previously enrolled in one MOOC, 44 in two, 195 in more than 2, and 252 are missing values.

**Fig 1:**
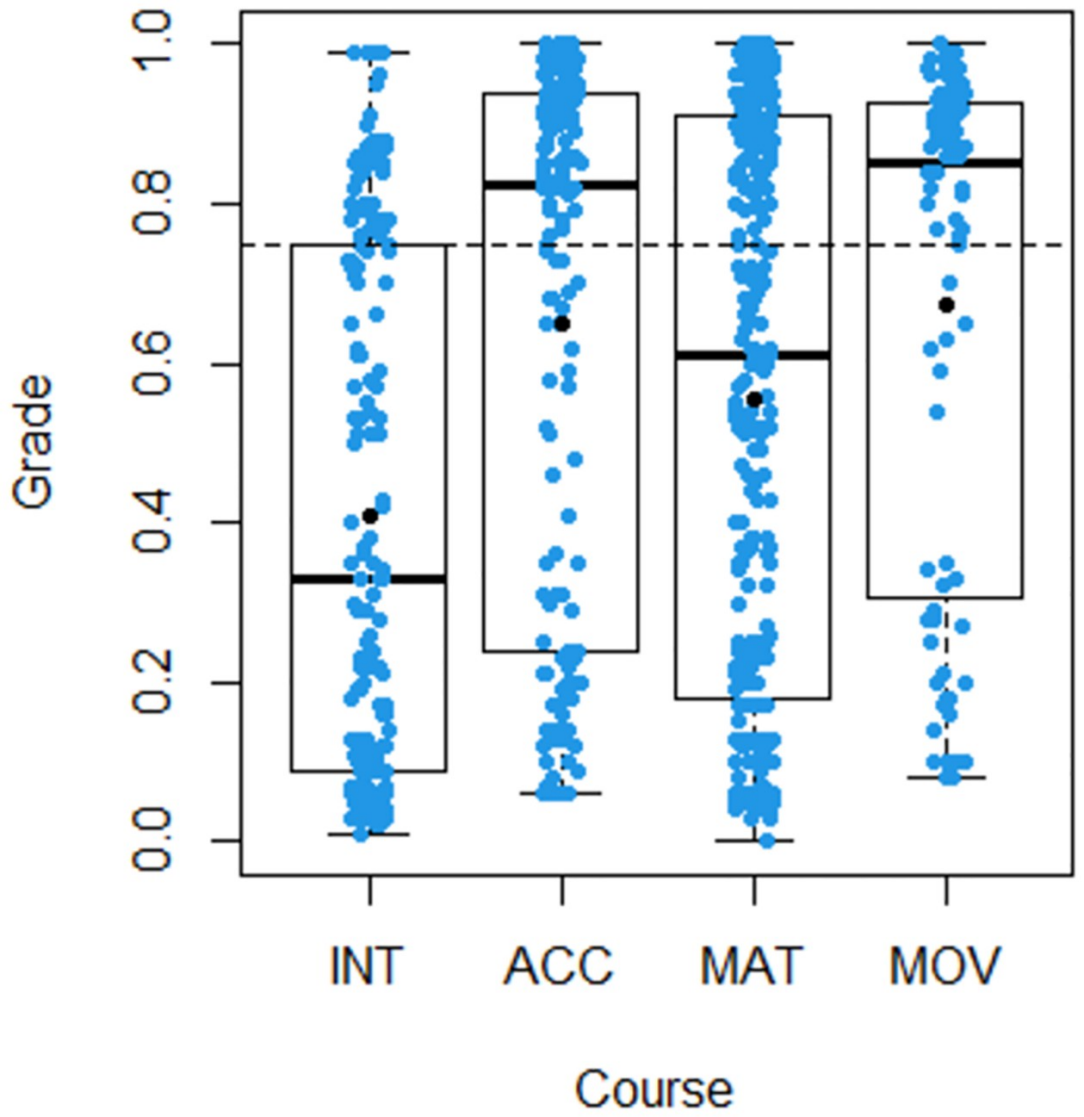
Boxplot of grade by course.

**Fig 2:**
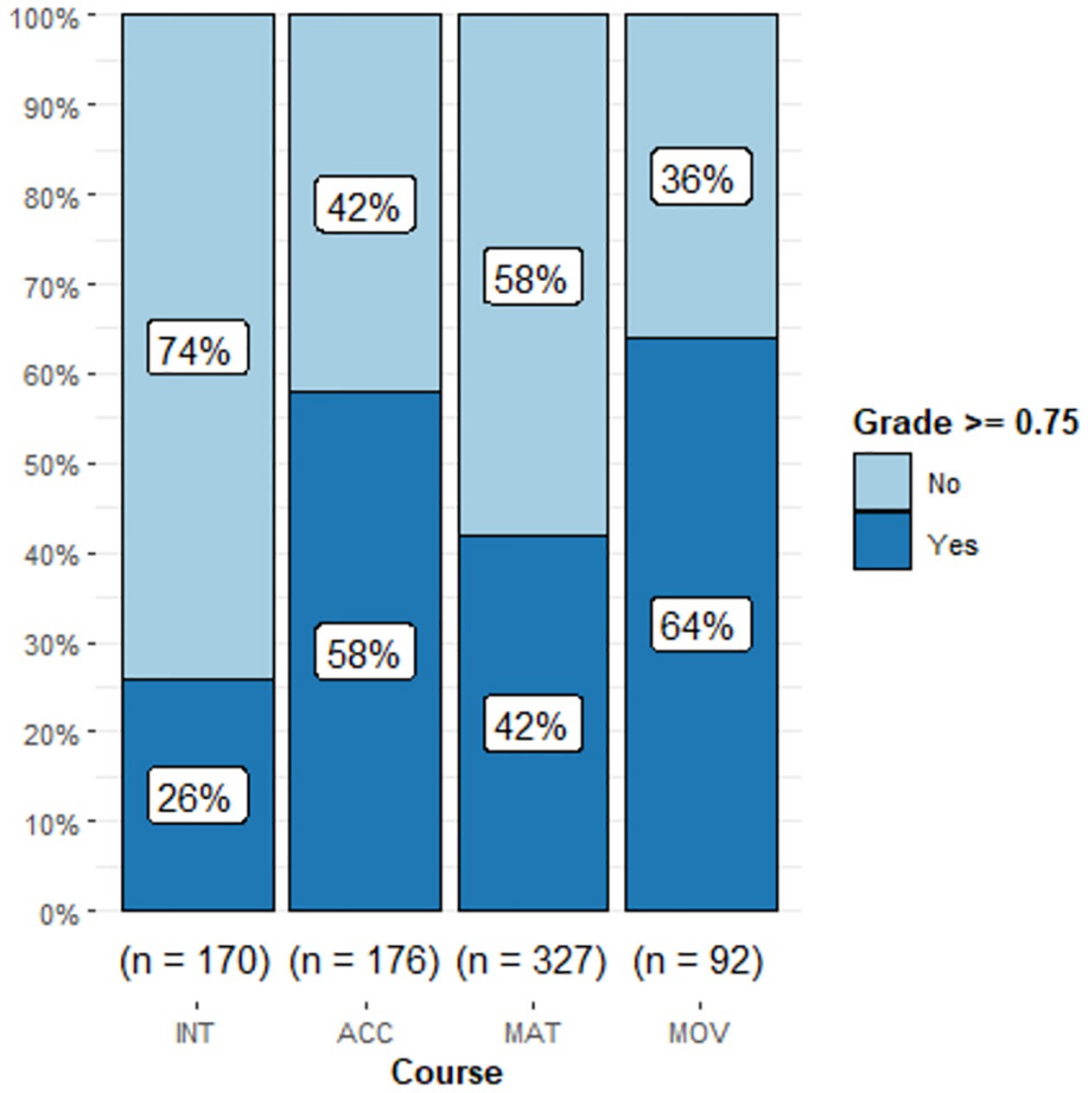
Stacked barplot of Grade≥0.75 by course.

In terms of performance per MOOC, we show for each course the boxplot of Grade (Fig 1) and the stacked barplot for the classification in terms of reaching/not reaching good performance, as defined above (Fig 2). It is observed that students of the INT course did worst in terms of mean (0.408), median (0.330) and percentage of good performance (26%), in spite of being the course with the highest participation. The variability of Grade was similar among courses as can be seen in Fig 1.

**Fig 3:**
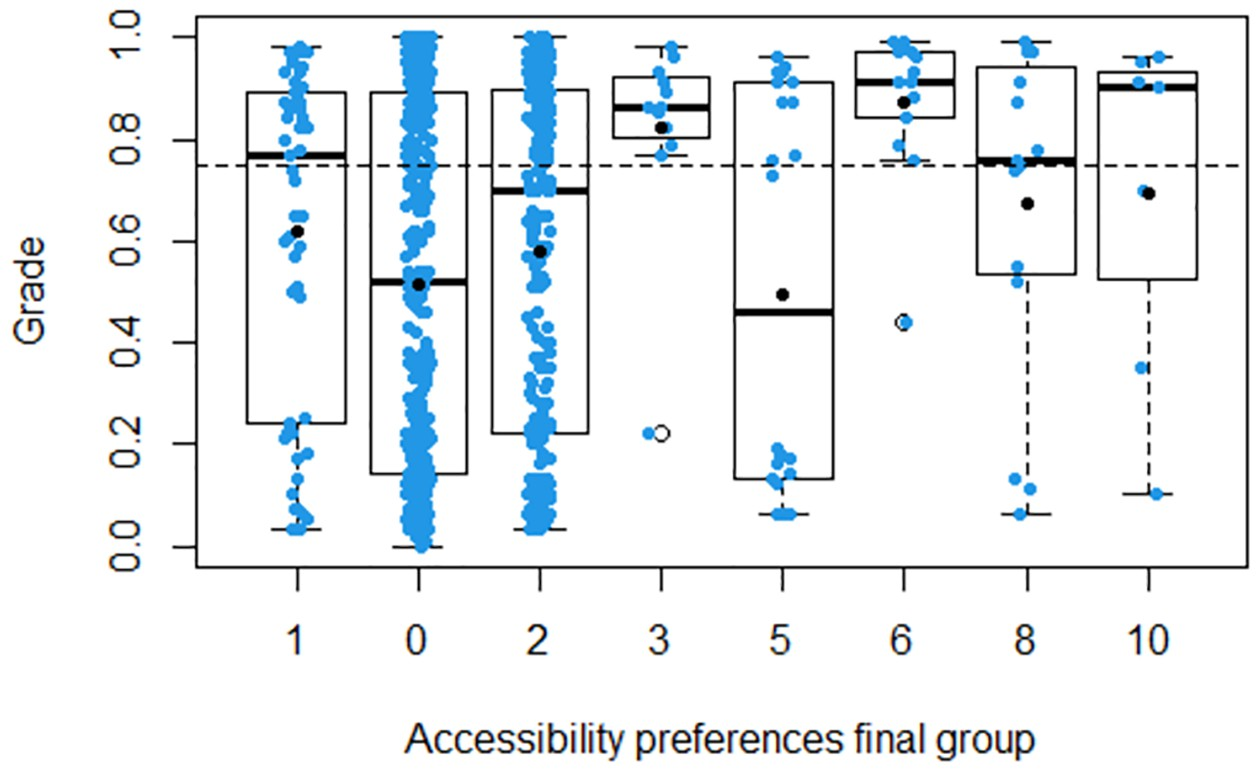
Boxplot of Grade by accessibility preferences group.

**Fig 4:**
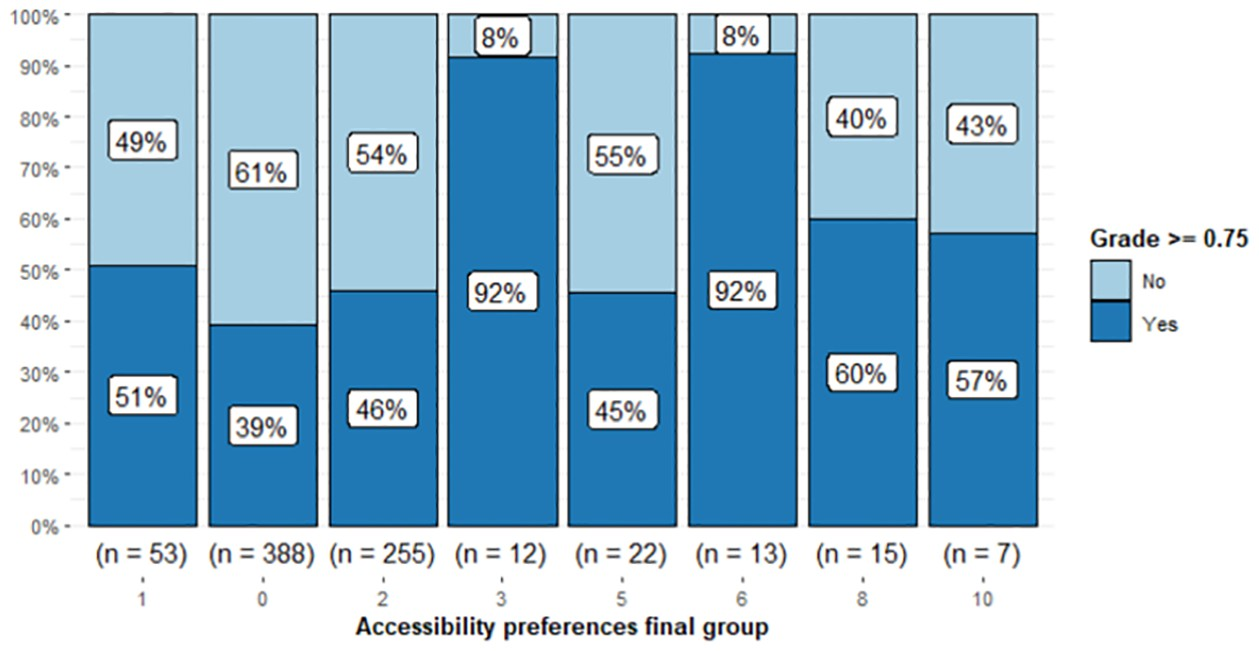
Stacked barplot of Grade≥0.75 by accessibility preferences group.

In terms of performance per group of accessibility preferences, we show for each group the boxplot of Grade (Fig 3) and the stacked barplot for the classification in terms of good performance or not (Fig 4). Regarding Groups 3, 5, 6, 8 and 10, Fig 3 shows that:

- The best performance was achieved by Groups 6 and 3. Note that the first quartiles are above the line of good performance in the Figure.
- The worst performance was achieved by Group 5. Note that it has the lowest first quartile.

Regarding the students in Group 0, they performed similarly to Group 5, while Groups 1 and 2 performed worse than Groups 8 and 10 but better than Group 5. It is worth mentioning that the two groups with the best performance (Groups 6 and 3) had less variability in Grade than the other groups (their interquartile ranges, *Q*_3_ − *Q*_1_, are the lowest).

It is observed in Fig 4 that the threshold value of 0.75 in Grade yields a good separation among the groups with less variability (Groups 6 and 3) and more variability (the other groups).

Regarding Groups 3, 5, 6, 8 and 10, it is observed that in terms of good performance (see Fig 4):

- The best groups were Groups 6 and 3. Note that for each of these two groups, 92% of the students reached a good performance.
- The worst group was Group 5, where only 45% of the students reached a good performance.

Regarding the students in Groups 0 and 2, they performed similarly to Group 5, while Group 1 performed worse than Groups 8 and 10 but better than Group 5.

### 3.2 Multilevel multiple regression analysis

Regarding the two main explanatory variables (Course and Group), the Shapiro-Wilk test indicates that the outcome variable (Grade) cannot be assumed normally distributed with respect to each category of the explanatory variables, therefore, a multilevel multiple logistic regression is used considering good performance or not as the outcome variable.

**Fig 5:**
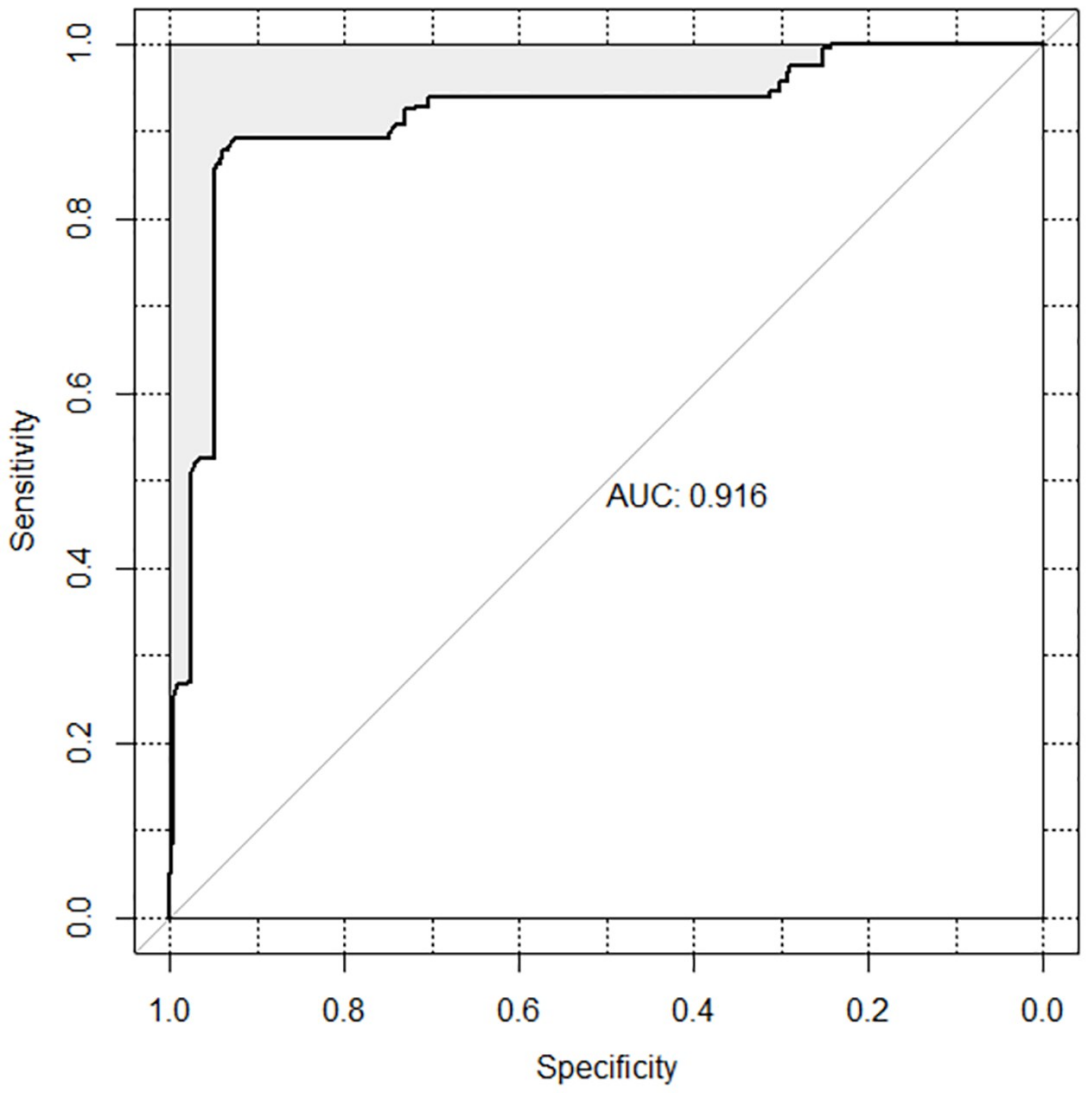
ROC curve associated with the multilevel multiple logistic regression fit.

Given that the results show that the INT course is clearly the most difficult one for students, a new explanatory variable will be considered, the result of re-coding the Course variable into a new variable, Course.INT, with two categories: INT and ACC+MAT+MOV. When performing the multilevel multiple logistic regression, INT and Group 1 will be taken as reference categories. In addition to Course and Group, the inclusion of demographic information as explanatory variables has been considered in case they were significant with the outcome variable, according to a bivariate analysis based on Fisher’s exact test. Since the two demographic variables that turned out to be significant in the bivariate analysis were Gender (*p*-value = 0.0055) and Sector (*p*-value = 0.0168), in the following we show the distribution of Gender and Sector against both Course.INT and Group (see Tables 5 and 6, respectively). However, the variables Gender and Sector did not turn out to be significant in the multivariate model, so the regression fit of the final model shown in Table 7 only includes Course and Group, the two main explanatory variables. The goodness of fit of this model, in terms of ROC curve, is given in Fig 5, with an AUC of 0.916 (95% CI: 0.8941–0.9388), which yields a very good model.

**Table 5:** Distribution of Gender according to Course.INT and Group.

Course.INT	0	0	1	1
Gender	Female	Male	Female	Male
Group				
1	14 (46.67%)	16 (53.33%)	10 (55.56%)	8 (44.44%)
0	75 (57.25%)	56 (42.75%)	21 (75.00%)	7 (25.00%)
2	115 (64.97%)	62 (35.03%)	35 (60.34%)	23 (39.66%)
3	1 (11.11%)	8 (88.89%)	1 (33.33%)	2 (66.67%)
5	10 (55.56%)	8 (44.44%)	1 (25.00%)	3 (75.00%)
6	2 (20.00%)	8 (80.00%)	1 (33.33%)	2 (66.67%)
8	5 (55.56%)	4 (44.44%)	4 (66.67%)	2 (33.33%)
10	0 (0.00%)	5 (100.00%)	1 (100.00%)	0 (0.00%)

**Table 6:** Distribution of Sector according to Course.INT and Group.

Course.INT	0	0	0	1	1	1
Sector	Private	Public	Others	Private	Public	Others
Group						
1	19 (63.33%)	9 (30.00%)	2 (6.67%)	6 (37.50%)	10 (62.50%)	0 (0.00%)
0	62 (51.24%)	58 (47.93%)	1 (0.83%)	12 (50.00%)	12 (50.00%)	0 (0.00%)
2	87 (51.48%)	78 (46.15%)	4 (2.37%)	28 (50.00%)	27 (48.21%)	1 (1.79%)
3	6 (75.00%)	1 (12.50%)	1 (12.50%)	1 (50.00%)	1 (50.00%)	0 (0.00%)
5	14 (82.35%)	3 (17.65%)	0 (0.00%)	3 (75.00%)	1 (25.00%)	0 (0.00%)
6	5 (50.00%)	5 (50.00%)	0 (0.00%)	2 (66.67%)	1 (33.33%)	0 (0.00%)
8	5 (62.50%)	3 (37.50%)	0 (0.00%)	3 (75.00%)	1 (25.00%)	0 (0.00%)
10	5 (100.00%)	0 (0.00%)	0 (0.00%)	1 (100.00%)	0 (0.00%)	0 (0.00%)

**Table 7:** Multilevel multiple logistic regression fit.

Fixed effects			
	*β* (se)	*p*-value	OR (95% CI)
Intercept	-0.832 (0.375)	0.0267	0.435 (0.209, 0.908)
Course.INT (1-level variable)			
INT (reference)	0	-	1
ACC+MAT+MOV	1.425 (0.251)	1.34e-08	4.158 (2.543, 6.799)
Group (2-level variable)			
1 (reference)	0	-	1
0	-0.922 (0.377)	0.0144	0.398 (0.190, 0.832)
2	-0.522 (0.382)	0.1722	0.593 (0.281, 1.255)
3	2.601 (1.234)	0.0351	13.482 (1.199, 151.532)
5	-0.688 (0.653)	0.2925	0.503 (0.140, 1.809)
6	2.618 (1.188)	0.0275	13.701 (1.336, 140.493)
8	0.337 (0.744)	0.6505	1.401 (0.326, 6.016)
10	0.034 (1.035)	0.9736	1.035 (0.136, 7.865)
Random effects			
2-level variance	0.787		
VP coefficient	0.193		
MOR	2.331		

VP, variance partition; MOR, median odds ratio.

In terms of the results given in Table 7, Course and Group effects are observed. More specifically, the course category ACC+MAT+MOV is statistically different from the reference category INT (*p*-value = 1.34 × 10^−8^), with the category ACC+MAT+MOV being the one positively associated with a “good result” (*OR* = *e*^1.425^ = 4.158, 95%*CI* = (2.543, 6.799)). It is important to note that the smallest effect would be of size 2.543, which would correspond to an increase of 154.3% in the odds of getting a good result.

Once the course effect is taken into account:

- In relation to RQ1, the categories associated with groups 0, 3 and 6 are statistically different from the reference category defined by group 1 (*p*-value = 0.0144, 0.0351 and 0.0275, respectively), observing a negative association with a good result in the group 0 (*OR* = *e*^−0.922^ = 0.398, 95%*CI* = (0.190, 0.832)), and a positive association in group 3 (*OR* = *e*^2.601^ = 13.482, 95%*CI* = (1.199, 151.532)) and in group 6 (*OR* = *e*^2.618^ = 13.701, 95%*CI* = (1.336, 140.493)). In spite of the huge width of their confidence intervals, which is due to the small sample sizes in those groups, it is worthwhile mentioning that the smallest effect would be 1.199 and 1.336, which would correspond to an increase of 19.9% and 33.6% in the odds of getting a good result. Regarding the smaller effect on the outcome, we observe that it is given by group 0, with the smallest decrease of 1 − 0.832 = 16.8% in the odds of getting a good result.
- Regarding RQ2, when group 5 is taken as reference, the groups 3 and 6 show positive significant effects on the outcome (*OR* = *e*^3.289^ = 26.817, 95%*CI* = (2.019, 356.260); and *OR* = *e*^3.305^ = 27.254, 95%*CI* = (2.244, 330.935), respectively).

For the sake of illustrating the use of the model, five cases of students will be described next (see Table 8 for the detailed calculations).

1. For a student of the reference category, i.e. enrolled in the INT course and that belongs to Group 1, the probability of achieving good performance is 30.32%.
2. For a student enrolled in any of the ACC, MAT or MOV courses, and who belongs to Group 1, the probability increases to 64.41%.
3. For a student enrolled in any of the ACC, MAT or MOV courses, and who belongs to Group 3 (Group 6), the probability increases again to 96.06% (96.12%).
4. Considering the smallest effect, a student that is enrolled in ACC, MAT or MOV, and belongs to Group 1 has a probability of achieving good performance of 52.53%.
5. For the case that the student is enrolled in ACC, MAT or MOV but belongs to Group 3 (Group 6), the probability is 57.02% (59.65%).

**Table 8:** Detailed calculations of illustrative examples.

Example	Percentage
1	1/(1 + *e*^−(−0.832)^) = 30.32%
2	1/(1 + *e*^−(−0.832+1.425)^) = 64.41%
3	1/(1 + *e*^−(−0.832+1.4251+2.601)^) = 96.06%
	(1/(1 + *e*^−(−0.832+1.4251+2.618)^) = 96.12%)
4	1/(1 + *e*^−(−0.832+*log*(2.543))^) = 52.53%.
5	1/(1 + *e*^−(−0.832+*log*(2.543)+*log*(1.199))^) = 57.02%
	(1/(1 + *e*^−(−0.832+*log*(2.543)+*log*(1.336))^) = 59.65%)

## 4 Discussion

As for participation, 765 out of the 2714 students (i.e. 28%) who enrolled filled in at least one of the graded assessment activities of the course, see Table 4. This result is in line with other studies’, such as [41]. Furthermore, participation was lower in the case of students who did not take the accessibility preferences survey (Group 0, 18%) than in the groups who completed the survey (the immediately higher participation was for Group 8, 54%). This could be explained by the lower level of commitment of Group 0 with the course, in general.

**Fig 6:**
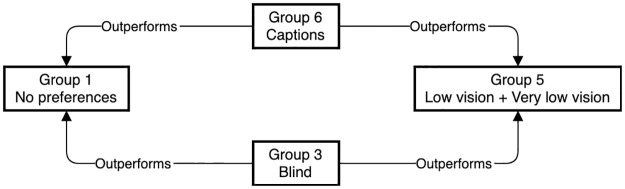
Comparison of group performance with statistically significant differences.

As for the grade, once the effect of the course has been eliminated, and the performance of the students who obtained good scores (above 0.75) has been analyzed, we can observe significant differences among groups of accessibility preferences (RQ2), see Subsection Multilevel multiple regression analysis. Both the group of blind students (Group 3) and the group of students who use captions (Group 6) perform better than the group of students with no accessibility preferences (Group 1). Furthermore, Group 3 and Group 6 perform significantly better than Group 5 (students with low vision + students with very low vision). Fig 6 shows the groups with statistically significant differences in performance. These results suggest that, in MOOCs complying with applicable accessibility and inclusion principles, some groups of students with disabilities have a good learning performance (RQ1). In some cases, i.e. blind students and students who use captions, they outperform their peers with no accessibility preferences (RQ2). In the MOOC realm there are no published studies relating to the performance of learners with disabilities taking up MOOCs [26, 36]. However, research indicates that some groups of students with disabilities perform worse than all students in online, formal education, see [25, 27, 39]. In particular [25], found specific groups of students with disabilities that showed lower pass rates and poorer grades than non-disabled students, namely students with mental health difficulties, students with restricted mobility, students with unseen or other disabilities, and students with dyslexia or other specific learning difficulties. Our results cannot be compared with theirs, since student groups are defined differently in both works: according to the type of disability (in their case), and according to the accessibility preferences or their needs when using digital learning materials (in ours). This leads to several differences in the formed groups: [25] establishes groups of students that aren’t included explicitly in our classification, namely: Restricted mobility; Restricted manual skills (difficulty handling items); Impaired speech; Dyslexia or other specific learning difficulties; Mental health difficulties; Personal care support; Fatigue or pain; Unseen disabilities (e.g. diabetes, epilepsy or asthma); Autistic spectrum disorder. Another difference derived from the approach to group students is that we make a difference for students: 1) using a screen reader (i.e., blind students) and students with low vision or with very low vision, all of them included in their “Blind or partially sighted” group; 2) users of captions and users of sign language, all of them included in their “Deaf or hard of hearing” group.

Moreover, it should be stressed that our results have been obtained in MOOCs training in Universal Design, and on top of that the platform and the contents comply with applicable accessibility standards. These features may have had a positive influence on the motivation of students with disabilities, who frequently face severe accessibility barriers in MOOCs, as described above. This claim could be in line with the results in [26], where learners with disabilities are particularly interested in taking up MOOCs in order to determine if they can study at a higher educational level and get flexibility and free education.

As could be foreseen, there are differences in the performance of groups with different accessibility needs and preferences (RQ2). Complying with widely accepted accessibility standards does not mean that all barriers are removed for all disability types or individuals. One salient example of this fact is that the group comprising both the students with low vision and the students with very low vision (Group 5) showed a negative association with a good result, when compared with blind students (Group 3) and with users of captions (Group 6), see Subsection Multilevel multiple regression analysis. How could the different attainments between these two groups with visual disabilities be explained? First, it needs to be considered that Group 5 consists of two raw groups of students, each of them using different strategies to overcome accessibility barriers. On the one hand, there are students with low vision (Raw Group 4) who only need an enhanced font size/contrast, and that therefore cope with the interaction by: 1) using accessibility features available in most of the mainstream browsers, and 2) relying completely on their sight. On the other hand, students with very low vision (Raw Group 5) make use of a screen magnifier, possibly enhanced with text-to-speech; these students cope with the MOOC by: 1) making use of an assistive product that requires specific training and skills, 2) relying on their sight and, in some cases, complementing it with their hearing. Fig 3 shows a high level of dispersion in the grades obtained by students in this group. The reason for defining this combined group was that neither of the low vision and very low vision groups had a sufficient number of students for the statistical analysis, when considered separately. Furthermore, existing literature shows differences in how blind students, on the one hand, and visually impaired students, on the other, make use of digital environments. [42] reviews different studies on this topic, which show that blind students apply recurring navigation tactics such as sequentially navigating the Web using arrow keys from top to bottom on a page; tabbing through links on the page; jumping from one section heading to another on a page; and using navigation functions of screen readers. In [43], one of the studies reviewed in [42], authors identified navigation tactics used by students of screen magnification software, which include exploring navigation bars, clicking links on navigation bars, using scroll bars, and exploring page contents. In [28], a group of users of both magnification software and text-to-speech experienced more usability problems than other groups of people with different accessibility preferences (including blind students), when making use of an elearning service.

We are aware that our study may have several limitations. To begin with, the set of preferences that students could choose from included only sensory alternatives for accessing digital learning resources. Options related to mobility aspects of interaction with digital resources were not considered. Furthermore, our results could be biased because the participation in the survey of accessibility preferences was voluntary. This survey is so far the only method we have to gather this type of information, key to our study. As similar studies pointed out [44], students taking the survey are less likely to drop out. However, it does not imply that the derived information is not usable. It indicates that survey participants, either if they declare to have accessibility preferences or not, could be considered as committed learners. Another limitation, mentioned in Section 3.1, is that preliminary results showed small sample sizes in several raw groups, which led us to combine groups with similar accessibility characteristics. Moreover, the topic of all the MOOCs had to do with Design for All, plus the fact that the courses were publicized as accessible for people with disabilities, may attract and motivate students differently, depending on whether they have a disability or not.

## 5 Conclusions and future work

We can conclude that taking accessibility into consideration from the outset, which implies applying UDL and WCAG 2.1, allows for the good performance of students with certain accessibility needs and preferences (RQ1). More specifically, and according to the regression model of our study, both the group of blind students and the group of students who use captions showed a positive, significant association with good performance in our courses, in comparison with students with no accessibility needs or preferences (RQ2). What is more, the group of students with low vision or with very low vision showed a negative, significant association with good performance in our courses, in comparison with the group of blind students and with the group of students who use captions (RQ2). The result for other groups is not conclusive, a potential reason being the low number of participants in some of them.

Furthermore, when designing student-centered MOOC services, the adoption of the appropriate surveys and user modeling techniques can represent an alternative to the needs assessment processes available in formal online education programs. These processes should be focused on gaining knowledge about how each student copes with digital learning resources, and not on her/his generic type of disabilities, or their medical causes. However, there are several groups that have not been targeted in our study, e.g., students with specific mobility or strength limitations, students with cognitive impairments, or students with mental illness.

In addition, complying with well-established web accessibility standards may not benefit all students with disabilities in the same proportion. Further research is needed about each user group, its retention and performance, and, more specifically about its coping tactics in the MOOC context, as well as what specific resources could be provided to them in this widely available, but mostly informal type of elearning. More specifically, our results recommend that usability tests of both the Open edX MOOC platform and courses used in this research should be undertaken for at least two student groups, namely students with low vision and students with very low vision. The reasons for these recommendations are the following: 1) when the course is controlled by using a regression model, these students analyzed as a single group are associated negatively to good performance in comparison to blind students and students using captions; 2) previous studies report underperformance of students with very low vision when using elearning services. Moreover, a closer look should be taken to the needs of students using sign language, and of students with mixed visual and hearing impairments, as well as to the appropriate accommodations that should be provided to them.

As examples of what other avenues of research should be further explored, participation and retention of students with disabilities is one of them. Another topic is the need of UDL-based frameworks for evaluating the appropriateness of MOOCs for different groups of students with disabilities in line with [18]. Likewise, we are aware that grades are only part of the complete picture of MOOC learning, as there are other ways to benefit from MOOCs that do not necessarily require completion [45, 46]. Moreover, learners’ motivations need to be investigated, as suggested in [47]. Finally [48], reviewed the issues that elearning systems present for students with cognitive disabilities, where there is a lot of improvement to be made.

## Supporting information

S1 Data Data file. This file contains the data collected during the learning experience.
(CSV)
